# Vegetarian-style dietary pattern during adolescence has long-term positive impact on bone from adolescence to young adulthood: a longitudinal study

**DOI:** 10.1186/s12937-018-0324-3

**Published:** 2018-02-28

**Authors:** Elham Z. Movassagh, Adam D. G. Baxter-Jones, Saija Kontulainen, Susan Whiting, Michael Szafron, Hassan Vatanparast

**Affiliations:** 1 College of Pharmacy and Nutrition, University of Saskatchewan, 104 Clinic place, Saskatoon, SK S7N 2Z4 Canada; 2 College of Kinesiology, University of Saskatchewan, 87 Campus Drive, Saskatoon, SK S7N5B2 Canada; 3 School of Public Health, University of Saskatchewan, 104 Clinic place, Saskatoon, SK S7N 5E5 Canada

**Keywords:** Dietary patterns, Vegetarian, Adolescence, Bone mineral content, Bone mineral density, Young adulthood

## Abstract

**Background:**

The amount of bone accrued during adolescence is an important determinant of later osteoporosis risk. Little is known about the influence of dietary patterns (DPs) on the bone during adolescence and their potential long-term implications into adulthood. We examined the role of adolescent DPs on adolescent and young adult bone and change in DPs from adolescence to young adulthood.

**Methods:**

We recruited participants from the Saskatchewan Pediatric Bone Mineral Accrual Study (1991–2011). Data from 125 participants (53 females) for adolescent analysis (age 12.7 ± 2 years) and 115 participants (51 females) for adult analysis (age 28.2 ± 3 years) were included. Bone mineral content (BMC) and areal bone mineral density (aBMD) of total body (TB), femoral neck (FN) and lumbar spine (LS) were measured using dual-energy X-ray absorptiometry. Adolescent dietary intake data from multiple 24-h recalls were summarized into 25 food group intakes and were used in the principal component analysis to derive DPs during adolescence. Associations between adolescent DPs and adolescent or adult BMC/BMD were analyzed using multiple linear regression and multivariate analysis of covariance while adjusting for sex, age, the age of peak height velocity, height, weight, physical activity and total energy intake. Generalized estimating equations were used for tracking DPs.

**Results:**

We derived five DPs including “Vegetarian-style”, “Western-like”, “High-fat, high-protein”, “Mixed” and “Snack” DPs. The “Vegetarian-style” DP was a positive independent predictor of adolescent TBBMC, and adult TBBMC, TBaBMD (*P* < 0.05). Mean adolescent TBaBMD and young adult TBBMC, TBaBMD, FNBMC and FNaBMD were 5%, 8.5%, 6%, 10.6% and 9% higher, respectively, in third quartile of “Vegetarian-style” DP compared to first quartile (*P* < 0.05). We found a moderate tracking (0.47–0.63, *P* < 0.001) in DP scores at individual levels from adolescence to adulthood. There were an upward trend in adherence to “Vegetarian-style” DP and an downward trend in adherence to “High-fat, high-protein” DP from adolescence to young adulthood (*P* < 0.01).

**Conclusion:**

A “Vegetarian-style” DP rich in dark green vegetables, eggs, non-refined grains, 100% fruit juice, legumes/nuts/seeds, added fats, fruits and low-fat milk during adolescence is positively associated with bone health.

## Background

Peak bone mass (PBM) attained by the end of adolescence is an early determinant of osteoporosis risk in older populations [1]. During adolescence, bone linear growth, and subsequent mineral deposition increase substantially [2]. The greatest rate of growth in height during this time is termed as peak height velocity (PHV). The PHV is considered as one of the main indicators of somatic maturation, the stage during which males and females are at a comparable sexual development milestone [3]. More than 39% of total body PBM is acquired during a 5-year period around PHV, and around 99% is attained by 6 years after attainment of PBM [4]. This suggests that modification of the factors that contribute to PBM attainment during adolescence might impact the risk of osteoporosis later in life [1].

Nutrition is an important modifiable factor, which could influence bone accrual, maintenance, and loss during one’s lifetime [1, 5]. Diet is a complex combination of nutrients and dietary components that correlate or interact with each other. Even though the separate role of key nutrients, or foods, on bone health has been reported previously, these associations might be confounded by any change in the other dietary components. Dietary pattern (DP) approaches describe and quantify the whole diet and consider contributions from various dietary aspects [6]. Findings from DP studies could complement those from studies of single nutrients and foods on bone accrual and may be translated into public health recommendations, which better suit real world dietary habits.

In adults and elderly, several studies have investigated the association between DPs derived by an exploratory method, mainly factor analysis, and bone health [7–20]. However, little is known about the DPs influencing bone health during adolescence [21–24], and their potential long-term implications. Therefore, longitudinal studies that follow participants from adolescence to adulthood are of immense importance because they could bridge the current gap in knowledge.

The objectives of our study are: 1) to examine the association between adolescent DPs and adolescent and young adult bone measurements including total body (TB), femoral neck (FN) and lumbar spine (LS) bone mineral content (BMC) and areal bone mineral density (aBMD), and 2) to evaluate the stability of DPs from adolescence to young adulthood. We hypothesized that a “healthy” DP, with an emphasis on higher intake of fruits, vegetables would be beneficial for adolescence and young adulthood bone health; and DPs remain relatively stable over time from adolescence to young adulthood.

## Methods

### Participants

We recruited participants from the Saskatchewan Pediatric Bone Mineral Accrual Study (PBMAS) (1991–2011). The mixed longitudinal design of the study has been described in detail elsewhere [3, 4, 25]. In brief, the PBMAS cohort consists of 251 individuals (133 girls and 118 boys; aged 8 to 15 years) recruited from two elementary schools in the city of Saskatoon between 1991 and 1993 who were subsequently followed with annual follow-ups until 2011. There were two four-year breaks in annual measurements: one between 1997 and 2002 and one between 2005 and 2010. The ages of the participants at the final follow-up were between 24 to 32 years. At each measurement occasion, participants underwent dual-energy X-ray absorptiometry (DXA) scans for bone and body composition. Anthropometry, dietary intake, and physical activity were also assessed at each measurement point.

For the present study, the first measurement within the age of PHV ± 2 years was considered as the adolescent measurement. For most participants (*n* = 105), the data collected during 1992 or 1993 were included in the analysis as adolescent data. The data collected during 2010 or 2011 were included in the analysis as young adult data. We included data from 125 participants (age 12.7 ± 2 years, 53 females) for adolescent analysis (cross-sectional) and 115 participants (age 28.2 ± 3 years, 51 females) for adolescence to young adult analysis (longitudinal). All participants or their parents provided informed written consent. Ethics approval was obtained from the University of Saskatchewan and Royal Hospital advisory boards on ethics in human experimentation [25].

### Dietary intake

The dietary intakes of participants were assessed using 24-h recalls. To determine accurate estimates of portion sizes, participants had access to pictures of foods. Adolescent dietary intakes were assessed by two to four (mostly three) 24-h recalls collected over a year and were analyzed using the Canadian compatible nutrition assessment software: NUTS Nutritional Assessment System, version 3.7 (Quilchena Consulting Ltd., Victoria, BC, 1988) to estimate the daily total energy and nutrient intakes. The average dietary intakes per day during the study year were stratified with the other annual measurements during the same year. To include in DP analysis, first, we converted quantities of all consumed foods and beverages into grams per day; then, all items were assigned into 25 pre-defined non-overlapping food groups, manually, based on similar nutrient content or culinary usage of them (Table 1). Young adult dietary intakes were assessed using one 24-h recall and estimates of total energy and nutrient intakes were obtained using Food Processor version 8.0 and its revisions (ESHA Research Inc., Salem, Ore, 2003).

**Table 1:** Food groupings used for principal component analysis to identify dietary patterns during adolescence

Food groups	Food items
Dark green vegetables	Asparagus, green beans, broccoli, lettuce, green pepper, seaweed, spinach, mixed greens, snow peas
Eggs	Eggs
Non-refined grains	Whole grains and partially whole grains (60%) mostly cereals, mixed granola/grain bar, cracker, oat flakes, wheat germ, whole wheat breads, puffed wheat, brown and wild rice, popcorn, barley
Fruit juice 100%	Apple cider, apple, lemon, lime, orange juice canned or bottled, unsweetened cranberry, etc.
Legumes, nuts and seeds	Beans (black, kidney, lima, navy, small white, soy), chickpeas, hummus, tofu, brazil nuts, coconut, almond, hazelnuts, walnuts, cashew, peanuts, mixed nuts, pecans, peanut butter, sunflower seeds
Added fats	Saturated fats such as butter, margarine, meatless bacon bits and coconut oil, and unsaturated fats such as vegetable oil, cooking oil, mayonnaise, olive oil, pesto
Fruits	All fresh and dried fruits, canned fruits (not sweetened), avocado, olives
Low-fat milk	1%, skim, rice beverage, soy beverage
Fruit drinks	Fruit juice (sweetened), fruit drinks, iced tea
Refined grains	Refined cereals, white bread, white rice, refined pasta, noodles, pop corns, pie crust, pizza pop
Cream	Sour cream, cream (10%, whipped or low fat)
Poultry	Chicken and turkey
Processed meats	Burger patties (beef, ham, chicken, etc.), sausages, bacon, canned meat, dry ribs, fried chicken, nugget
High-fat milk	2%, whole or almond milk
Tomato	Tomato and its products
Red meat	Beef, ham, pork, bison (ground, loin, rib, steak, stew, fried, pot roast, balls, loaf, chop)
Cheese	Cheddar, cream cheese, feta, gouda, mozzarella, parmesan, Swiss, cottage, ricotta, cheese sauce
Yogurt	Yogurt (plain, vanilla or fruit)
Desserts and sweets	Sweet baked products, milk desserts, jelly, chocolate, sugar, jam, syrups, honey and candies
Fish and seafood	Fish, shrimp, lobster, mussels, pickerel, prawns, scallops
Dressings, sauces, gravy	Gravy, dressings, Caesar, French, ranch, Italian, 1000 island, Alfredo, blue cheese, chip dip, Greek, honey garlic, white sauce, sandwich spread, tartar, teen, sundried tomato
Vegetables, others	Carrots, snap beans, cabbage, cauliflower, celery, cucumber, garlic, mushroom, pepper, squash, bean sprouts, beets, onion, eggplant, radish, zucchini, potato, green peas, corn, sweet potato and soups
Chips & fries	Potato chips, fries, corn chips, nacho, hash brown
Soft drinks	Soft drinks (sugar-sweetened or diet)
Others	Salt, spices, seasonings, additives, pickles (dill, beet), low fat sauces (mustard, hot, soy, teriyaki), vinegar

### Bone mineral content and areal density

Adolescent and young adult BMC and aBMD of TB, FN and LS (L1–L4) were measured using DXA (Hologic QDR 2000, Hologic, Inc., Waltham, MA, USA) in the array mode; and analysis was conducted using enhanced global software version 7.1 [26]. To minimize operator-related variability in the scan analysis over the years, the same trained person analyzed all scans. The TB scans were analyzed using software version 5.67A and scans of the FN and LS were analyzed using software version 4.66A. The in vivo coefficients of variations, which represent short-term precision, were comparable to the values from other studies employing the QDR 2000 in the array mode (0.60, 0.91 and 0.61 for TB, FN, and LS BMC, respectively).

### Physical activity

Physical activity was defined as sports, games, or dance that makes you breathe hard, makes your legs feel tired, or makes you sweat. The physical activity questionnaire (PAQ) was used to assess adolescent physical activity during spare time in the previous 7 days by rating nine items in elementary schools or eight items in high schools (excluding the item regarding activity at recess) scored on a five-point scale [27]. Six of these questions were related to scaling the level of different activities in physical education classes, recess, lunch, right after school, in the evenings and on the weekend. Other three questions were asking about the frequency of physical activity during each day, the number of hours spent for watching TV, and describing the whole week activity from low to very high activity levels [28]. The average score derived from each PAQ ranged from one to five, with higher scores indicating higher levels of physical activity. To assess young adult physical activity, PAQ was modified to a 7-item questionnaire including more age-relevant activities. The school-day structure of questions was replaced with a day section structure (i.e., morning, after lunch, before supper, evening) in the PAQ for adults [28]. The PAQ was administered three times a year during first 3 years of study and two times a year thereafter. The average PA scores derived from PAQs collected during each year were aligned with the other annual measurements [26].

### Anthropometry and age of PHV

Weight and stature were measured following standard protocols for each participant while wearing lightweight clothing and no shoes [25]. To control for somatic maturity, the age of PHV for each participant was estimated. The process for determining PHV has been described elsewhere [26]. In brief, whole-year height increase velocity was computed using serial measurements of height for each participant by age. Using a cubic spline procedure, a growth curve was fitted to each individual’s annual height velocities (GraphPad Prism Version 3.00) and the age of PHV was determined from the estimated growth curve [26].

### Statistical analysis

The DPs were identified using factor analysis via principal component analysis (PCA). The PCA aggregates the food groups into a smaller number of the distinct factors based on inter-correlation between them [6, 29]. To achieve a simpler structure with higher interpretability, orthogonal rotation (Varimax option) was applied. Overall, 11 factors were extracted using PCA with an eigenvalue > 1 accounting for 66% of the total variance in all food group intakes. Based on the breakpoint in scree plot, we retained 5 major factors (accounting for almost 40% of the total variance) for further evaluation and re-ran the analysis with a five-factor solution. Factor loadings represent the correlation between food groups and the factors (Table 2). The absolute value represents the strength of the correlation. A positive loading shows a direct association and a negative loading shows an inverse association between the food group intake and DP score. Food groups with a factor loading ≥0.35 or ≤ − 0.35 were considered informative for interpretation of DPs in our study. Regression scores for each DP were calculated using the regression scores option in SPSS. Calculating regression scores enhances the validity of DP scores and reduces the probability of biased estimates of the true scores [30].

**Table 2:** Factor loading of food groups in five dietary patterns identified by principal component analysis during adolescence, in participants of Pediatric Bone Mineral Accrual Study (PBMAS), *n* = 125^1^

	Factor Loadings for Dietary Patterns				
	Vegetarian-Style	Western-Like	High-Fat, High-Protein	Mixed	Snack
Dark green vegetables	**0.64**	0.02	− 0.00	0.07	− 0.22
Eggs	**0.63**	− 0.18	0.23	− 0.05	− 0.15
Non-refined grains	**0.54**	− 0.13	− 0.11	0.10	0.20
Added fats	**0.41**	**0.39**	− 0.03	− 0.04	− 0.00
Fruits	**0.40**	0.24	− 0.16	0.13	0.23
Others	− 0.28	0.03	0.08	0.08	0.04
Fruit drinks	0.00	**0.73**	− 0.04	− 0.03	0.04
Refined grains	0.06	**0.66**	0.21	− 0.10	− 0.03
Cream	− 0.06	**0.55**	− 0.01	0.13	− 0.02
Poultry	− 0.27	**0.41**	− 0.04	− 0.10	**0.40**
Processed meats	− 0.05	**0.35**	− 0.12	0.01	− 0.09
High-fat milk	− 0.12	− 0.17	**0.74**	− 0.04	0.18
Tomato	0.22	0.30	**0.59**	− 0.14	− 0.34
Red meat	− 0.07	− 0.05	**0.52**	0.14	− 0.07
Low-fat milk	**0.35**	0.03	**− 0.48**	− 0.01	− 0.16
Legumes, nuts, and seeds	**0.45**	0.11	**0.47**	− 0.09	0.06
Cheese	0.03	0.12	0.06	**0.72**	**− 0.36**
Yogurt	− 0.11	0.04	− 0.12	**0.61**	0.19
Desserts and sweets	− 0.18	− 0.05	0.23	**0.59**	0.08
Fish and seafood	0.24	− 0.10	−0.08	**0.52**	− 0.13
Fruit juice 100%	**0.46**	0.02	− 0.04	**0.49**	0.18
Dressings, sauces, gravy	0.09	− 0.30	0.24	0.08	**0.64**
Vegetables, others	− 0.03	0.22	0.06	− 0.03	**0.58**
Chips & fries	− 0.03	− 0.09	− 0.02	0.00	**0.40**
Soft drinks	0.00	− 0.02	− 0.16	− 0.20	0.20
% Of variance explained	9.2	8.5	7.8	7.7	6.7

^1^Factor loadings ≥0.35 or ≤ − 0.35 have been presented

The bold numbers represent the foods with significant positive or negative loading in each pattern

Descriptive statistics for all bone variables (TBBMC, TBaBMD, FNBMC, FNaBMD, LSBMC, LSaBMD), and covariate variables (age, the age of PHV, height, weight, physical activity score and total energy intake) were presented as mean ± SD in adolescence and young adulthood. We used independent Student’s t-test to compare variables of interest between females and males. Multiple linear regression using stepwise procedure were conducted to evaluate associations between adolescence DP and adolescence bone measurements. To assess the long-term impact of DPs on the bone, we also ran the same modeling with adolescent DP scores as predictor variables, and young adulthood bone measurements as outcome variables. All models were adjusted for sex, the age of PHV, age, height, weight, physical activity score and total energy intake. Covariates measured during adolescence and young adulthood were used in the adolescence and young adulthood models, respectively.

Comparisons of the mean adolescence or young adult bone variables across the quartile categories of adolescent DP score were conducted via a multivariate analysis of covariance (MANCOVA) (with a Bonferroni adjustment for multiple comparisons) while adjusting for scores of the other four DPs (as continuous variables), sex, age of PHV, age, height, weight, physical activity score and total energy intake.

To evaluate the stability of DPs from adolescence to young adulthood, we calculated applied DP scores during adolescence and young adulthood, based on the factor loadings for 25 food groups in five DPs derived during adolescence. To control for the overall increase in consumption of food groups by age from adolescence to young adulthood, we computed the consumed amount (g) per 1000 kcal of total energy intake for each food group. Then, these energy-adjusted intakes were multiplied by their corresponding factor loading in each DP and were summed up as the DP score. We standardized adolescence and young adulthood DP scores for mean and standard deviation of adolescence DP scores in our sample. Then we calculated tracking coefficients using generalized estimating equations (GEE). Tracking coefficient represents how position of participants in a study population distribution is maintained from baseline to the last follow-up [31]. We regressed adolescence standardized DP scores (independent variable) against young adulthood standardized DP scores (dependent variable) while adjusting for chronological age as the time-dependent variable, and sex and age at adolescence as time-independent variables. The ß coefficient of adolescence standardized DP scores takes values between 0 to 1, representing no tracking and strong tracking, respectively. The ß coefficient for chronological age indicates the change in DP score as z-score or SD for each year increase in age.

The DP analysis and all other statistical analyses were performed using SPSS software, version 24.0 (SPSS, Chicago, lL, USA). *P* < 0.05 was considered significant.

## Results

The characteristics of the study population during adolescence and young adulthood are shown in Table 3. Our estimated mean ± SD follow-up period from adolescence to young adulthood was 15.5 ± 3.4 years. The first factor, labeled as “Vegetarian-style” DP, was rich in dark green vegetables, eggs, non-refined grains, 100% fruit juice, legumes, nuts and seeds, added fats, fruits and low-fat milk (including non-dairy milk). The second factor, a “Western-like” DP was associated with higher intakes of fruit drinks, refined grains, cream, poultry and processed meats. The most significant characteristic of the third factor, “high fat, high protein” DP, was high positive loadings for High-fat milk, tomato, red meat and legumes, nuts and seeds and a negative loading for low-fat milk. The fourth factor, a “Mixed” DP, was characterized by a high intake of yogurt, cheese, desserts and sweets, fish and seafood and 100% fruit juice. Dressings and sauces, vegetables (excluding dark green vegetables), chips and fries and poultry had high positive loadings and cheese had a negative loading in the fifth factor, labeled a “Snack” DP (Table 2).

**Table 3:** Descriptive characteristics during adolescence and young adulthood by sex^1^

	Females	Males	Total
Adolescence	*n* = 53	*n* = 72	n = 125
Biologic age^2^ (year)	0.2 ± 1.7	−0.1 ± 1.8	0.0 ± 1.7
Age (year)	12.0 ± 1.8	13.2 ± 1.8*	12.7 ± 1.9
Age of PHV (year)	11.8 ± 0.8	13.2 ± 0.9	12.6 ± 1.2
Physical activity (score)	2.9 ± 0.7	3.0 ± 0.6	3.0 ± 0.7
Total energy intake (kcal/d)	1714 ± 461	1978 ± 615*	1867 ± 569
Height (cm)	153 ± 11	162 ± 14**	158 ± 13
Weight (kg)	46.0 ± 14	52.4 ± 14*	49.8 ± 14
TBBMC (g)	1402 ± 452	1751 ± 612**	1604 ± 575
TBaBMD (g/cm^2^)	0.87 ± 0.10	0.94 ± 0.12*	0.91 ± 0.11
FNBMC (g)	3.3 ± 0.8	4.1 ± 1.0**	3.8 ± 1.0
FNaBMD (g/cm^2^)	0.73 ± 0.13	0.81 ± 0.13*	0.77 ± 0.13
LSBMC (g)	35.8 ± 13.4	40.8 ± 16.0	38.7 ± 15.1
LAaBMD (g/cm^2^)	0.76 ± 0.15	0.75 ± 0.14	0.76 ± 0.14
Young adulthood	*n* = 51	*n* = 64	n = 115
Biologic age^2^ (year)	16.1 ± 3.5	15.0 ± 3.3	15.5 ± 3.4
Age (year)	27.9 ± 3.4	28.3 ± 3.4	28.2 ± 3.4
Physical activity (score)	2.3 ± 0.6	2.3 ± 0.7	2.3 ± 0.6
Total energy intake (kcal/d)	1823 ± 698	2823 ± 1235**	2401 ± 1151
Height (cm)	166 ± 7	179 ± 7**	174 ± 9
Weight (kg)	70.7 ± 16	87.0 ± 14**	80.3 ± 16
TBBMC (g)	2286 ± 321	3020 ± 413**	2706 ± 523
TBaBMD (g/cm^2^)	1.12 ± 0.09	1.22 ± 0.10**	1.18 ± 0.11
FNBMC (g)	4.3 ± 0.7	5.6 ± 0.8**	5.0 ± 0.9
FNaBMD (g/cm^2^)	0.86 ± 0.10	0.95 ± 0.128**	0.91 ± 0.12
LSBMC (g)	62.0 ± 12.6	76.2 ± 12.8**	70.3 ± 14.5
LSaBMD (g/cm^2^)	1.04 ± 0.12	1.06 ± 0.12	1.05 ± 0.12

*Abbreviations*: *aBMD* areal bone mineral density, *BMC* bone mineral accrual, *FN* femoral neck, *LS* lumber spine, *PBMAS* the pediatric bone mineral accrual study, *PHV* the peak height velocity, *TB* total body

^1^Values are Mean ± SD. *P* values were obtained using independent samples Student’s *t* test. ^*^Different from females, *P* < 0.01. ^**^Different from females, *P* < 0.001

^2^Biologic age is calculated as chronologic age minus the age of PHV

After controlling for covariates (sex, age of PHV and adolescent age, height, weight, physical activity score and total energy intake), multiple linear regression showed that the “Vegetarian-style” DP was a positive independent predictor of adolescent TBBMC (β =35.2, *P* = 0.025; R^2^ = 0.84) and young adult TBBMC (β = 55.8, *P* = 0.021; R^2^ = 0.78), TBaBMD (β = 0.016, *P* = 0.041; R^2^ = 0.67). No other adolescent DP was found to be an independent predictor for any of the adolescent or young adult bone variables.

Comparison of adolescent or young adult bone variables across adolescent DP score quartiles showed that, those in the third quartile of “Vegetarian-style” DP had 5.7%, 8.5%, 6%, 10.6% and 9% higher adolescent TBaBMD (Table 4), and young adult TBBMC, TBaBMD, FNBMC and FNaBMD (Table 5), respectively, compared to their peers in the lowest quartile, after adjusting for covariates and other four DP scores as continuous variables.

**Table 4:** Adolescence bone variables across the quartile groups of each dietary patterns derived during adolescence^1^

	Dietary pattern score quartiles^2^				*P* value
	Quartile1 (n = 31)	Quartile2 (*n* = 31)	Quartile3 (n = 31)	Quartile4 (*n* = 32)	
Vegetarian-style					
TBBMC	1555.34 ± 33.15	1579.43 ± 31.58	1649.63 ± 32.15	1634.61 ± 32.15	0.18
TBaBMD	0.88 ± 0.01^a^	0.90 ± 0.01^a,b^	0.93 ± 0.01^b^	0.91 ± 0.01^a,b^	0.025
FNBMC	3.64 ± 0.01	3.69 ± 0.01	3.86 ± 0.01	3.79 ± 0.01	0.31
FNaBMD	0.75 ± 0.01	0.78 ± 0.01	0.80 ± 0.01	0.76 ± 0.01	0.22
LSBMC	37.08 ± 1.18	39.68 ± 1.18	39.03 ± 1.19	38.9 ± 1.19	0.52
LSaBMD	0.73 ± 0.01	0.77 ± 0.01	0.77 ± 0.01	0.75 ± 0.01	0.20
Western-like					
TBBMC	1612.62 ± 33.61	1623.64 ± 31.58	1594.61 ± 32.25	1588 ± 33.32	0.86
TBaBMD	0.91 ± 0.01	0.91 ± 0.01	0.91 ± 0.01	0.90 ± 0.01	0.74
FNBMC	3.78 ± 0.11	3.68 ± 0.11	3.8 ± 0.12	3.8 ± 0.12	0.92
FNaBMD	0.79 ± 0.01	0.76 ± 0.01	0.77 ± 0.01	0.78 ± 0.01	0.82
LSBMC	39.21 ± 1.22	39.52 ± 1.14	38.18 ± 1.21	37.79 ± 1.21	0.73
LSaBMD	0.76 ± 0.01	0.76 ± 0.01	0.76 ± 0.01	0.75 ± 0.01	0.93
High-fat, high-protein					
TBBMC	1630.53 ± 32.15	1597.64 ± 32.14	1586.65 ± 32.15	1603.71 ± 34.28	0.82
TBaBMD	0.91 ± 0.01	0.90 ± 0.01	0.90 ± 0.01	0.91 ± 0.01	0.92
FNBMC	3.79 ± 0.01	3.88 ± 0.01	3.67 ± 0.01	3.68 ± 0.01	0.40
FNaBMD	0.77 ± 0.01	0.79 ± 0.01	0.77 ± 0.01	0.77 ± 0.01	0.74
LSBMC	38.91 ± 1.19	38.91 ± 1.19	39.08 ± 1.28	37.79 ± 1.28	0.91
LSaBMD	0.74 ± 0.01	0.76 ± 0.01	0.77 ± 0.01	0.76 ± 0.01	0.77
Mixed					
TBBMC	1580.01 ± 32.38	1608 ± 30.28	1657 ± 30.73	1572 ± 32.75	0.22
TBaBMD	0.89 ± 0.01	0.91 ± 0.01	0.92 ± 0.01	0.90 ± 0.01	0.24
FNBMC	3.79 ± 0.01	3.68 ± 0.01	3.88 ± 0.01	3.69 ± 0.01	0.41
FNaBMD	0.77 ± 0.01	0.77 ± 0.01	0.80 ± 0.01	0.76 ± 0.01	0.50
LSBMC	38.68 ± 1.21	37.77 ± 1.10	40.86 ± 1.10	37.31 ± 1.21	0.16
LSaBMD	0.75 ± 0.01	0.75 ± 0.01	0.78 ± 0.01	0.75 ± 0.01	0.37
Snack					
TBBMC	1587.11 ± 30.77	1639.27 ± 30.22	1590.44 ± 31.17	1601.11 ± 32.54	0.59
TBaBMD	0.90 ± 0.01	0.92 ± 0.01	0.90 ± 0.01	0.91 ± 0.01	0.37
FNBMC	3.81 ± 0.09	3.85 ± 0.09	3.68 ± 0.09	3.88 ± 0.09	0.43
FNaBMD	0.79 ± 0.01	0.78 ± 0.01	0.75 ± 0.01	0.78 ± 0.01	0.31
LSBMC	38.09 ± 1.12	41.05 ± 1.12	37.31 ± 1.12	38.44 ± 1.22	0.12
LSaBMD	0.76 ± 0.01	0.79 ± 0.01	0.73 ± 0.01	0.75 ± 0.01	0.055

*Abbreviations*: *aBMD* areal bone mineral density, *BMC* bone mineral accrual, *FN* femoral neck, *LS* lumber spine, *TB* total body

^1^Values are Mean ± SE. Mean adolescence bone variables were adjusted for sex and adolescent age of peak height velocity, age, height, weight, physical activity score, total energy intake and other four dietary pattern scores as continuous variables and were compared across quartiles of adolescence dietary pattern scores using MANCOVA with Bonferroni adjustment for multiple comparisons. Labeled means in a row without a common superscript letter differ, *P* < 0.05

^2^Participants in Quartile four have the highest adherence to the DPs in adolescence

**Table 5:** Young adulthood bone variables across the quartile groups of each dietary patterns derived during adolescence^1^

	Dietary pattern score quartiles^2^				*P* value
	Quartile1 (n = 29)	Quartile2 (*n* = 29)	Quartile3 (n = 29)	Quartile4 (*n* = 28)	
Vegetarian-style					
TBBMC	2592.38 ± 46.12^a^	2693.36 ± 46.12^a,b^	2813.68 ± 47.22^b^	2709.64 ± 49.25^a,b^	0.016
TBaBMD	1.14 ± 0.01^a^	1.18 ± 0.01^a,b^	1.21 ± 0.01^b^	1.18 ± 0.01^a,b^	0.017
FNBMC	4.69 ± 0.12^a^	5.02 ± 0.12^a,b^	5.19 ± 0.12^b^	5.08 ± 0.12^a,b^	0.042
FNaBMD	0.87 ± 0.02^a^	0.92 ± 0.02^a,b^	0.95 ± 0.02^b^	0.89 ± 0.02^a,b^	0.020
LSBMC	66.27 ± 1.91	71.75 ± 1.91	72.17 ± 2.04	68.91 ± 2.04	0.14
LSaBMD	1.00 ± 0.02	1.06 ± 0.02	1.08 ± 0.02	1.04 ± 0.02	0.09
Western-like					
TBBMC	2742.45 ± 47.45	2688.48 ± 47.62	2745.88 ± 48.22	2629.84 ± 48.24	0.28
TBaBMD	1.18 ± 0.01	1.17 ± 0.01	1.20 ± 0.01	1.15 ± 0.01	0.24
FNBMC	5.04 ± 0.11	4.91 ± 0.11	5.15 ± 0.12	4.90 ± 0.12	0.39
FNaBMD	0.91 ± 0.02	0.90 ± 0.02	0.93 ± 0.02	0.90 ± 0.02	0.71
LSBMC	70.10 ± 2.01	71.31 ± 1.90	71.34 ± 2.02	66.12 ± 2.02	0.25
LSaBMD	1.05 ± 0.02	1.05 ± 0.02	1.07 ± 0.02	1.01 ± 0.02	0.35
High-fat, high-protein					
TBBMC	2715.42 ± 48.68	2692.14 ± 50.25	2684.42 ± 48.58	2712.85 ± 47.85	0.96
TBaBMD	1.18 ± 0.01	1.18 ± 0.01	1.17 ± 0.01	1.18 ± 0.01	0.98
FNBMC	5.02 ± 0.11	5.20 ± 0.11	4.74 ± 0.11	5.08 ± 0.12	0.07
FNaBMD	0.90 ± 0.02	0.93 ± 0.02	0.88 ± 0.02	0.92 ± 0.02	0.27
LSBMC	67.90 ± 2.01	72.40 ± 2.11	68.60 ± 2.04	70.0 ± 2.04	0.45
LSaBMD	1.02 ± 0.02	1.07 ± 0.02	1.04 ± 0.02	1.06 ± 0.02	0.41
Mixed					
TBBMC	2713.25 ± 48.32	2700.38 ± 48.32	2721.25 ± 48.32	2668 ± 49.12	0.88
TBaBMD	1.17 ± 0.01	1.19 ± 0.01	1.18 ± 0.01	1.17 ± 0.01	0.78
FNBMC	5.11 ± 0.12	5.11 ± 0.12	4.90 ± 0.12	4.88 ± 0.12	0.28
FNaBMD	0.91 ± 0.02	0.93 ± 0.02	0.89 ± 0.02	0.89 ± 0.02	0.46
LSBMC	68.67 ± 2.02	70.18 ± 2.02	72.52 ± 2.02	67.52 ± 2.02	0.36
LSaBMD	1.04 ± 0.02	1.06 ± 0.02	1.07 ± 0.02	1.02 ± 0.02	0.50
Snack					
TBBMC	2673.32 ± 45.45	2780.77 ± 47.32	2652 ± 46.87	2699 ± 47.35	0.24
TBaBMD	1.17 ± 0.01	1.20 ± 0.01	1.17 ± 0.01	1.17 ± 0.01	0.58
FNBMC	5.01 ± 0.12	5.01 ± 0.12	4.80 ± 0.13	5.07 ± 0.13	0.64
FNaBMD	0.92 ± 0.02	0.90 ± 0.02	0.88 ± 0.02	0.92 ± 0.02	0.41
LSBMC	68.22 ± 1.9	72.04 ± 2.02	68.11 ± 2.02	70.51 ± 2.02	0.45
LSaBMD	1.05 ± 0.02	1.07 ± 0.02	1.03 ± 0.02	1.04 ± 0.02	0.58

*Abbreviations*: *aBMD* areal bone mineral density, *BMC* bone mineral accrual, *FN* femoral neck, *LS* lumber spine, *TB* total body

^1^Values are Mean ± SE. Mean young adulthood bone variables were adjusted for sex and age of peak height velocity and young adult age, height, weight, physical activity score, total energy intake and other four adolescence dietary pattern scores as continuous variables and were compared across quartiles of adolescence dietary pattern scores using MANCOVA with Bonferroni adjustment for multiple comparisons. Labeled means in a row without a common superscript letter differ, *P* < 0.05

^2^Participants in Quartile 4 have the highest adherence to the DPs in adolescence

Tracking coefficients for standardized scores of five DPs and change in the score by age from adolescence to young adulthood are presented in Table 6. The greater tracking coefficients show the higher stability of DPs at the individual level. Since DP scores have been standardized for the baseline DP scores, ß coefficient for age variable represents the amount of change in z-score. Overall, energy-adjusted scores increased for “Vegetarian-style” and decreased for “High-fat, high-protein” DP, from adolescence to young adulthood (Table 6).

**Table 6:** Tracking coefficients and change in score by age for dietary patterns derived during adolescence^1^

	Tracking dietary patterns			Change in dietary pattern score		
	ß (adolescence score)	95% CI	*P* value	ß (age)	95% CI	*P* value
Vegetarian-style	0.59	0.48, 0.71	< 0.001	0.026	0.00, 0.04	0.008
Western-like	0.47	0.40, 0.53	< 0.001	− 0.008	− 0.029, 0.012	0.42
High-fat, high-protein	0.51	0.41, 0.60	< 0.001	− 0.019	−0.034, − 0.005	0.009
Mixed	0.54	0.39, 0.69	< 0.001	−0.003	−0.033, 0.028	0.85
Snack	0.63	0.55, 0.70	< 0.001	−0.003	−0.023, 0.018	0.80

*Abbreviations*: *CI* confidence intervals

^1^Generalized estimating equations was used for modeling association between adolescence and adulthood standardized and energy-adjusted dietary pattern scores while controlling for sex, age, and age at adolescence; *n* = 115. Tracking coefficient (ß coefficient for adolescent dietary pattern) shows how participants maintained their position in the study population distribution, between adolescence and young adulthood. Tracking coefficient for age represents z score change in dietary pattern score from adolescence to young adulthood

## Discussion

In our prospective study, we found that a “Vegetarian-style” DP rich in dark green vegetables, eggs, non-refined grains, 100% fruit juice, legumes, nuts and seeds, added fats, fruits and low-fat milk during adolescence was associated positively with adolescent TBBMC and TBaBMD. We also found that participants who had higher adherence to the “Vegetarian-style” DP during adolescence had higher TBBMC, TBaBMD, FNBMC and FNaBMD during young adulthood, average 15 years later. Tracking DP scores showed that participants moderately maintained their position in the study population distribution from adolescence to young adulthood, which means DPs were relatively stable over time. However, the overall adherence to “Vegetarian-style” DP increased from adolescence to young adulthood.

In the present study, the favorable effects of the “Vegetarian-style” DP were only observed in TB and FN bone measurements, but not in LS bone. This might be due to the different proportions of cortical and trabecular bone compartments in different skeletal sites. The trabecular bone is the predominant bone compartment in LS, while TB and FN mainly contain cortical bone [32, 33]. Trabecular bone is metabolically more active than cortical bone and might be influenced by everyday changes in hormone or environmental factors. Hence adaptations in bone might last longer in cortical compared to trabecular bone [34].

Our study is unique as it evaluated the long-term impact of adolescent DPs on young adult bone. To our knowledge, there are only four studies that evaluated the DPs during adolescence in association with bone health [21–24]. Even though three of these studies were similar to our study in their prospective design (follow-up period ranged from 22 months to 6 years) [21, 23, 24], identified DPs are not directly comparable, because of the differences in DP approaches, food groupings and dietary habits and other characteristics of the study population [3, 6]. However, our findings of a positive association between “Vegetarian-style” DP and bone measurements are in accordance with the results from two studies which used reduced-rank regression (RRR) to derive DPs. The RRR has the advantage of deriving DPs associated with bone variables such as BMD and BMC [21] or intermediate factors such as protein, calcium, and potassium [2], as response variables. In Korean girls (aged 9–11 years, *n* = 198), the RRR-derived “fruits, nuts, milk beverages, eggs, and grains” DP was associated positively and “egg and rice” DP was associated negatively with BMC gain after 22 months [21]. Also, a higher intake of low-fat dairy, whole grains, and vegetables, as components of a DP rich in protein, calcium and potassium in Australian adolescents (aged 14 years, *n* = 1024) was associated with higher BMD and BMC at age 20 years [24]. Overall, higher intakes of fruit and vegetables, milk and alternatives, nuts and grains were the common components in all DPs which determined to be beneficial for bone [2, 21]).

Our results are also in line with the findings from previous DP studies in adults and elderly populations suggesting that a high intake of fruit and vegetables, whole grains, poultry and fish, nuts and legumes and low-fat dairy products labeled as “healthy” DP is beneficial for bone health [7–10, 12–14, 16, 17]. Vegetables, fruits, and 100% fruit juices are rich in potassium, magnesium, vitamins C, K and folate and carotenoids [35]. Potassium and magnesium may contribute to acid-base balance [35] and calcium metabolism [36, 37] to prevent bone loss. Vitamin C, carotenoids, and other antioxidants may affect bone health through their antioxidant properties, which suppress osteoclast activity [38, 39]. Vitamin C also acts as a cofactor for osteoblast differentiation and collagen formation [38, 40]. Vitamin K also plays a role in bone matrix formation where mineralization happens [41]. Low-fat milk and its alternatives are the main contributors of calcium and magnesium in diet [42], which have a structural role in bone health [43]. Calcium from vegetable sources also has been shown to be positively effective in bone maintenance in older ages [44]. They are also a source of protein, vitamin D, vitamin B12, zinc and riboflavin [42]. An adequate protein intake is essential for bone matrix formation and maintenance. Eggs, legumes, nuts and seeds, as meat alternatives, are good sources of protein [45]. Dietary fiber from non-refined grains and other plant sources might also have a beneficial impact on bone through decreasing glycemic load and inhibiting hyperinsulinemia which in turn prevents urinary calcium loss induced by insulin [46]. Added fats including, mainly, butter, margarine, and mayonnaise as one of components of the “Vegetarian-style” DP might play a role in providing adequate dietary energy for adolescents during their growth spurt, when they are consumed along with other components of “Vegetarian-style” DP. Lower intake of meat seems to be beneficial, as this seems to be one of the key differences between “Vegetarian-style” DP and other four DPs. Taken together, the “Vegetarian-style” DP represents a combination of beneficial nutrients and dietary components with potential synergic or interacting effects. Therefore no single nutrient or dietary components could be pointed out as the one responsible for the beneficial impact of the DP on bone.

Our study has several strengths. This is the first study that evaluated DPs during adolescence in association with young adult bone health. In our sample, all participants during young adulthood had their PBM confirmed by a plateau in bone mineral accrual curve, representing a steady status of bone [4]. We also controlled for somatic maturity by including the age of PHV as a covariate in our models. Adolescent dietary intake data were collected using multiple, mostly three, 24-h recalls over a year for each participant, which is preferred to food frequency questionnaires [47], the method used by most previous studies. In addition, we analyzed the impact of the whole diet, instead of a single food or nutrient, on bone.

The main limitation of our study was the small sample size (*n* = 125 for adolescent analysis, and *n* = 115 for young adult analysis), which did not allow us to run the separate analysis for females and males or run other DP approaches such reduced-rank regression method. Small sample size also limited us from adding more covariates in the model such as young adult DPs, smoking status, oral contraceptive use or reproductive history (in females). Even though we did not control the models for young adult DPs, we assessed change in DPs from adolescence to young adulthood to overcome this limitation. Two further limitations of our study are reliance on only one 24-h recall in young adulthood and using two different nutrient assessment systems from adolescence to young adulthood. However, our focus was food group intake and these two systems were only used to measure total energy intake.

## Conclusions

Our results suggest that a diverse and well-balanced DP, rich in dark green vegetables, eggs, non-refined grains, 100% fruit juice, legumes, nuts and seeds, added fats, fruits and low-fat milk during adolescence has a beneficial impact on bone health during adolescence and this positive impact on bone accrual can be carried into young adulthood. Further population-based studies are needed to confirm our findings and generalize these results to other populations.

## Abbreviations

aBMD: areal bone mineral density
BMC: Bone mineral content
DP: dietary pattern
DXA: dual-energy X-ray absorptiometry
FN: femoral neck
LS: lumbar spine
MANCOVA: multivariate analysis of covariance
PAQ: physical activity questionnaire
PBM: peak bone mass
PBMAS: Pediatric Bone Mineral Accrual Study
PCA: principal component analysis
PHV: peak height velocity
TB: total body
